# Assessment and Conceptualization of Perceived Competence in Ice Hockey: A Scale Development and Validation Study

**DOI:** 10.1177/00315125231170915

**Published:** 2023-04-26

**Authors:** Vincent Huard Pelletier, Jean Lemoyne

**Affiliations:** 1 Department of Human Kinetics, 14847 Université du Québec à Trois-Rivières, Trois-Rivieres, QC, Canada; 2 Laboratoire de recherche sur le hockey de l’UQTR, 14847 Université du Québec à Trois-Rivières, Trois-Rivieres, QC, Canada

**Keywords:** psychometric development, youth sport, personal development, sport psychology, competence in sport

## Abstract

Perceived competence plays a crucial role in establishing environments favorable to individual development in youth sport. As most assessment tools of perceived competence are not sport-specific, they have limited usefulness for sport practitioners and researchers. This study had two-fold aims: (i) to develop a perceived competence assessment tool specific to ice hockey; and (ii) to examine its factorial structure and internal consistency. We first developed an initial 29-item version of this ice hockey competence self-report scale, using a group of ice hockey stakeholders and sports science experts to develop the items and a pilot sample of 42 hockey players to confirm test-retest reliability. Finally, we validated the scale among a cohort of 770 adolescent ice hockey players (*M* age = 14.78, *SD* = 1.60 years). Results from exploratory factor analysis (EFA) revealed that perceived competence in ice hockey was defined by six dimensions, with rejection of seven items. Confirmatory factor analysis (CFA) suggested that the 6-factor first order model was the best fit with the conceptualization of perceived competence in ice hockey (CFI = 0.938, RMSEA = 0.044). The final, 22-item questionnaire now provides a reliable and valid measure of perceived competence in hockey for adolescent participants. It has promise for evaluating future interventions aiming to foster young athletes’ perceived confidence through sport.

## Introduction

The benefits associated with participation in youth sport have been well documented. On the physical side, sport participation has been associated with positive changes in aerobic capacity, body composition and musculoskeletal health (Conn et al., 2011). From a public health perspective, a review by Lee et al. (2018) showed that youth sport increases players’ levels of engagement in physical activity, which results, in turn, in such health advantages as the prevention of long-term obesity prevention. Other long-term benefits of youth sport participation include improved general fitness (Cordingley, 2019). Involvement in sports also fosters the development of motor skills that promote long-term participation in varied types of physical activity (Loprinzi et al., 2012). For children and adolescents, sport participation has positively affected psychological outcomes and has suggested favorable social outcomes, such as better emotion regulation, quality relationships with peers and diminished depressive symptoms (Staurowsky, 2015); and the same themes have held for young adults (Sabiston et al., 2016). These benefits have extended to better overall mental health, with improved self-esteem, life skills, and/or social interactions (Eime et al., 2013). From this perspective, sports federations have implemented long-term models of athlete development that promote early sport participation (Stafford, 2005). In most sports, these models promote the acquisition of fundamental skills in the initial stages of participation (e.g., 6–10 years) and put more emphasis on sport-specific expertise later (e.g., 12–17 years). At the same time, most sport development models have encouraged creating environments that foster players’ enjoyment, autonomy, increased competence, and sense of accomplishment.

Organized sport is one of the most popular forms of physical activity among youth (Emmonds, 2021; Ifedi, 2008; Merkel, 2013), and, among winter sports, one of the most popular is ice hockey. Ice hockey has worldwide popularity, with more than 80 countries currently registered in the International Ice Hockey Federation (IIHF) database (IIHF, 2021). Hockey Canada, with more than 500,000 registered players, is the world’s largest ice hockey federation. Hockey Canada’s long-term development model integrates various player-centered areas of skill development, such as skating, stickhandling, tactics, decision-making and self-confidence, with a structured plan for coaches and stakeholders in the design of players’ developmental pathways from youth to adulthood (Hockey Canada, 2013). In fact, there is a long-term process for developing hockey players; the complexity of this sport might be why players tend to quit it by the end of adolescence. Among the numerous challenges associated with keeping young people engaged in any sport are time conflicts with other activities, lack of motivation, low perceived competence, and negative relationships with coaches (Balish et al., 2014).

### The Importance of Perceived Competence in Sport

In sport and exercise psychology, the concept of autonomy support has been extensively studied. Preston and Fraser-Thomas (2018) showed that coaches who try to develop their players’ abilities and successes face particular challenges in trying to create autonomy supportive environments, in which players’ personal improvements and engagement are highly valued. Establishing such environments requires enhancing young athletes’ motivation and desire to pursue their personal development through sport participation.

Self-determination theory (SDT; Deci & Ryan, 1980) is among the most frequently cited theoretical models for motivational development. This model stipulates that intrinsic motivation is reinforced when experiences in sport (or other domains) contribute to nourishing the participants’ sense of autonomy (e.g., making choices), relatedness (e.g., experiencing personal significance) and feelings of self-competence (e.g., feeling good about abilities). Since high levels of intrinsic motivation are associated with persistence in sport (Vallerand & Losier, 1999), it is crucial to reinforce young athletes’ positive perceptions about their own abilities. Thus, an individual’s perceived competence in a specific field of activity is related to their long-term engagement in it (Rottensteiner et al., 2015).

Barnett et al. (2008) showed that perceived sport competence of youth determines their later engagement in physical activities in late adolescence. Perceived competence refers to an individual’s positive perceptions of their abilities in a particular performance domain (Horn, 2004). Developed by Harter (1978), the theory of competence motivation suggests that young people who consider themselves proficient in a specific skill area tend to invest more energy to further improve their skills in that area. In line with SDT assumptions (and previous research), Harter proposed that skills improvement in a specific area of expertise should lead to higher perceived competence, which results, in turn, in higher autonomous motivation towards the activity (Babic et al., 2014; Côté & Vierimaa, 2014; Harter, 1978). There is substantial empirical support for the importance of perceived competence in sport in physical education settings (Tsang, 2007) and in different sport contexts. Thus, sport competence should be a key consideration within sport development programs (Bailey et al., 2013).

Perceived sport competence is one of four subdomains of the physical self-concept; others are physical strength, body attractiveness, and physical condition (Estevan & Barnett, 2018). Physical self-concept is itself a subdomain of the global self-concept (Estevan & Barnett, 2018). Bortoli et al. (2011) first linked perceived sport competence and current expertise, and he subsequently demonstrated that these two concepts interact to predict a healthy psychological state. The assessment of perceived competence among youth sport participants, especially with respect to their main athletic activity, is critical to understanding the quality of their sport experience a specific different developmental stages.

Traditionally, perceived sport competence has been measured in terms of general physical competence or with general athletic competence scales (Estevan & Barnett, 2018; Harter, 1985). However perceived competence has long been considered a multidimensional, hierarchical concept based on young athletes’ interpretations of their abilities in comparison with significant others (e.g., teammates) and/or performance feedback from their coaches and parents (Shavelson et al., 1976). According to Lemoyne, et al. (2015), adolescents with strong perceived sport competence are more inclined to be engaged in the corresponding type of activity. This association between perceived competence and active behaviors has been observed among elite swimmers (Marsh & Perry, 2005) and teenage gymnasts (Chanal et al., 2005). In this broad context, assessessment tools for perceived sport competence may need to be specific to separate sport activities. In fact, There is high value in developing a questionnaire to assess perceived competence that is specific to ice hockey; ice hockey calls for a vast repertoire of skills and abilities, and perceived competence in these skills is relevant for researchers who wish to analyze the outcomes of ice hockey participation on individual development (Estevan & Barnett, 2018; Huard Pelletier et al., 2020; Huard Pelletier & Lemoyne, 2022).

Since ice hockey competition has become international, numerous hockey development programs have prioritized providing positive experiences through sport. Assessing perceived competence among ice hockey players is relevant for many reasons: (a) knowing how young athletes perceive themselves in their sport can help coaches and program directors implement approaches that foster players’ self-enhancement within a favorable motivational climate; (b) developing a sport specific perceived competence instrument will advance new self-enhancement research efforts in the a specific sport; and (c) measuring perceived sport competence in ice hockey can help promote positive relationships between ice hockey stakeholders (coaches, players and parents). Indeed, different emphases across this triad of themes in the evaluation of players' skills may constitute become a source of conflict with negative consequences for a young person’s sport experience (Preston et al., 2020; Smoll et al., 2011), possibly contributing to sport attrition. There is a clear need to develop a specific ice hockey competence questionnaire for both ice hockey practitioners and researchers.

### Assessment of Perceived Sports Competence

There have been prior attempts to measure perceived competence in individual sports such as judo (Le Bars et al., 2009), gymnastics (Kipp & Weiss, 2013) and elite swimming (Marsh & Perry, 2005). These measures usually consisted of an adaptation of Harter’s 5-item Self-Perception Profile for Adolescents (Harter, 1988) or of the Perceived Competence subscale from the Intrinsic Motivation Inventory (McAuley et al., 1989). Marsh and Perry (2005) revealed that swimmers’ self-concepts were a valid indicator of their performance in international events. In ice hockey, Rottensteiner et al. (2015) verified and confirmed the important role of perceived competence on developing autonomous motivation and persistence in sport among 1962 adolescent Finnish players. Such results are encouraging and affirm that perceived sport competence is a key factor to consider in athletic development. However, a limitation of previous instruments is that most of them focused on a general conceptualization of sport competence (e.g., with survey items like, *I am good at sport*) that does not inform us as to how players perceive the expertise that is unique to their target sport. For example, since hockey involves a vast repertoire of skills and abilities, a player might see himself-herself simultaneously as a good offensive player (high perceived competence) but a poor defensive player (low perceived competence).

An example of a sport-specific instrument is the Perceived Game-Specific Soccer Competence Scale (PGSCS), developed by Forsman et al. (2016). This instrument was shown to be a valid, reliable, and practical questionnaire. Forsman et al. (2016) developed it with 1,321 adolescent soccer players. The PGSCS consists of 18 items that assesses three dimensions of game-related soccer skills (e.g., individual tactics, defensive play, offensive play). This scale was subsequently validated, and its three sub-dimensions were found to be significantly associated with intrinsic motivation and soccer-specific skills on the field (Forsman et al., 2016). The results of this study are promising and can perhaps be replicated in other international and multidimensional sports such as ice hockey.

### Objectives of this Study

In this study, we had two objectives: (a) to develop a questionnaire that measured perceived competence in ice hockey; and (b) to test the questionnaire’s construct validity, factorial structure, and other psychometric properties. We expected the concept of perceived sport competence in ice hockey to be multidimensional and correlated with general sport competence.

## Method

### Overview of the Study

**Figure 1.**
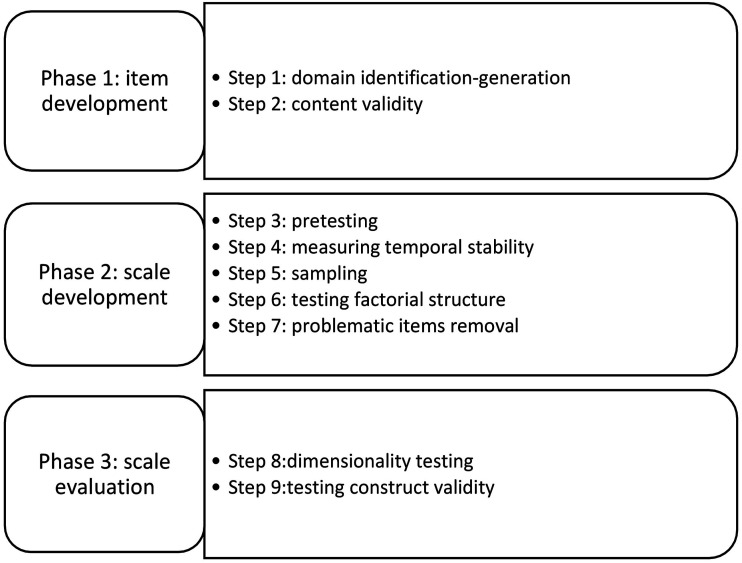
Overview of the Study.

The overall structure of this study followed recommendations of Boateng et al. (2018); we conducted it in three phases: (a) item development, (b) scale development and (c) scale evaluation. Phase 1 (item development) was conducted in two steps: domain identification-generation (step 1) and assessment of content validity (step 2). Phase 2 (scale development) involved pretesting the survey (step 3), measuring test-retest reliability of the instrument (step 4), establishing the minimal sample size for distributing the scale to a validation sample (step 5), testing the scale’s factorial structure (step 6), and removing problematic items (step 7). Phase 3 involved structural equation modeling and testing the dimensionality of the scale (step 8) and then verifying the scale’s construct validity through its association with a component of physical self-concept, global perceived sport competence (step 9). Due to needs for sanitary precautions amidst the uncertainty of COVID-19 risks at the time of data collection, we modified the normal order of steps proposed by Boateng et al. (2018) by moving test-retest reliability testing to phase 2, just before the determination of sample size and main distribution of the questionnaire for validation testing. We implemented this phase earlier due to the restrictions related to the COVID-19 pandemic, approaching only players who were proximal to the researchers’ institution. During the period when we decided to test the instrument’s test-retest reliability, ice hockey play had been banned in most regions of Quebec but was still possible in some other regions. With the season already ending, we proceeded with the test-retest; fortunately, the other regions were able to start playing again before the end of the season. Thus, while COVID-19 restrictions affected ice hockey play, they did not affect the distribution of the questionnaire. Figure 1

### Phase 1: Item Development

#### Domain Identification and Item Generation

As suggested by Haynes et al. (1995), we first defined the sport domain to ensure that all items generated measured the same construct. Since no existing perceived competence instrument has been directed toward ice hockey, we proposed an informal definition of ice hockey competence, and then, with the help of Hockey Quebec, we invited 25 ice hockey stakeholders (e.g., coaches, scouts, general managers, etc.) to identify key determinants of ice hockey competence. These stakeholders provided a list of email addresses of individuals with whom they had worked in the past. Twenty-three agreed to participate upon receipt of the first email, and two others were added, following a seven-day reminder period. This phase of the study (and those that follow) were conducted in the province of Quebec, an almost exclusively francophone territory; so, the instrument was developed in French. Once experts were informed about our definition of hockey competence and the procedures, we collected data by using a questionnaire with two broad questions: (a) *From your background as a player/coach/scout, what are the different components or dimensions that could determine a hockey player’s competence?* and (b) *In relation with each component that you have mentioned in question 1, could you identify specific actions that we could observe (by providing examples) to assess each component?* Participants had plenty of space to enter their responses and could do so in any format they wished (e.g., bullet points, continuous text, key words, etc.). To proceed with the content analysis (Braun & Clarke, 2006), we followed the first two steps proposed by L'Écuyer (1990). First, we proceeded to a preliminary reading of all the responses and created a list of the statements participants suggested. Next, we defined a classification unit for each statement to reduce their length, keeping only the essential parts and facilitating item elaboration. The goal was to achieve a statement-to-item ratio in the questionnaire of approximately 5:1, as recommended by Schinka et al. (2012), allowing the experts to ensure that the concept of ice hockey proficiency was addressed in all its facets, while developing a questionnaire of less than 30 questions to make it faster to complete.

#### Content Validity Observation

The original set of items in the proposed scale was reviewed by four pre-appointed scientific experts from different fields (e.g., sports science, psychology, psychometrics), each with at least five publications related to team sports. They revised the items based on the Delphi Method (Vernon, 2009). During this process, the experts had to accept, recommend modification, or reject the items. Next, the authors modified the targeted items before returning them to the experts for another evaluation round. The experts reviewed items independently and anonymously over a 10-day period, and both authors then took seven days to further modify the questionnaire before the next expert review round. We deemed the item set acceptable when three out of the four experts (75%) reached agreement that it was satisfactory (Langlands et al., 2008).

### Phase 2: Scale Development

#### Scale Pretesting and Test-Retest Reliability

Each part of this project was pre-approved by the researchers’ institutional ethics board (CER-21-275-07.04). All participants or their parents (for participants younger than 14 years old, the age considered legally capable of giving consent in the jurisdiction where the research was conducted) gave informed voluntary consent to participate. We sent invitations to complete the initial version of the questionnaire to three ice hockey teams whose head coaches had previously indicated participation interest. Following the program directors’ and head coaches’ permissions to meet players, we informed players about the project, and asked willing participants (or their parents for those younger than 14) to sign a consent form. We asked players to complete the initial version of the questionnaire on two separate occasions in an online web format (Qualtrics, Provo, UT), over a seven-day period. This seemingly short interval was justified by the participants’ age and the changing nature of their perceived competence during a sport season (Ingrell et al., 2016; Polit, 2014). At the end of the questionnaire, players could indicate the questions they misunderstood and suggest possible improvements. We evaluated test-retest reliability of the scale by assessing and interpreting the intraclass correlation (ICC) coefficient based on single measures (e.g., Time 1 with Time 2) using IBM SPSS for Windows, version 28 (IBM Corp., Armonk, N.Y., USA). In line with Kline’s (2016) recommendations, ICCs over 0.70 were interpreted as indicators of good test-retest reliability.

#### Sample Size and Survey Administration

We determined sample size by following recommendations for structural equation modeling (SEM; Kyriazos, 2018; Soper, 2022) in which a sample of at least 200, participants are deemed to be needed to achieve sufficient statistical power. For this phase, we recruited participants in collaboration with the provincial ice hockey federation, who agreed to send invitations directly to ice hockey program directors across the province. Program directors from 29 of 45 contacted organizations agreed and were contacted by members of our research staff for an information session, after which we distributed the online Qualtrics invitations to players from 54 teams. Participant eligibility criteria were to be aged between 12-17 years-old and a registered hockey player. This age bracket allowed us to include most players in the U13, U15 and U18 levels, corresponding to the ages of youth in Quebec high schools. We informed players about the project and obtained written consent from players and/or their parents (for players younger then 14 years old). A second sample of ice hockey players was recruited to conduct the third phase of this study. These participants were part of a national team selection camp and had previously agreed to participate in a research project (authors, 2022) with the authors of this manuscript and had completed the questionnaire as part of that project.

#### Factor Extraction and Item Reduction

We established the factorial structure with exploratory factor analysis (EFA), using Mplus software (version 8). According to Kline (2016, p.190), the EFA procedure allows the evaluation of associations between each item and its corresponding specific factor. In addition, EFA offered the option of specifying as many factors as possible. We estimated different factor structures to verify which was the most appropriate, by comparing models with 1 to 7 dimensions. We used the Geomin rotation for factor extraction, because of its advantages in further model estimation, especially because it considers the possibility of interrelations between factors (Hattori et al., 2017). We compared all models by calculating the Satorra-Bentler chi-square difference test (SBΔχ^2^), to identify which had a better fit (Satorra & Bentler, 2001). After identifying the best fitting model, we identified the appropriate factor structure by comparing each model; we verified factor loadings for each item (e.g., after rotation) and identified items that displayed factor loading that were higher than .40 (Costello and Osborne, 2005). We categorized “problematic” items when they cross loaded (> .40) on multiple factors or no factor at all and retired them from the subsequent analyses. Once we identified items as factors indicators, we calculated the McDonald’s omega coefficient (Ω) to estimate the internal consistency for each sub-scale. We interpreted coefficient higher than 0.70 as being satisfactory (Tavakol & Dennick, 2011).

### Phase 3: Scale Evaluation

#### Test of Dimensionality

As the second part of the analysis, we tested the factorial structure of the questionnaire. Considering the latent nature of the perceived competence construct, we conducted SEM analyses by testing subsequent models with Mplus (version 8) software: Model 1- Confirmatory factor analyses (CFA), Model 2- CFA with addition of relevant covariances (according to the La Grange test), Model 3-Second order CFA. In Model 1, we constrained item loading to their corresponding factor (e.g., no cross-loadings allowed), which suggested that we test the structural model by assuming that each factor was measured by a specific set of indicators, as identified in previous EFA analyses. Missing data were minimal (< 8%), and we proceeded to missing data handling by using the Mplus default option (e.g., expectation maximization). Model fit was interpreted by using the most common SEM fit indices and we used MLR estimator for each model. Because chi-square (x^2^) values are sample dependent, we put more attention to the Root Mean Squared Error of Approximation (RMSEA), the comparative fit index (CFI), and the Tucker Lewis Index (TLI). Hu and Bentler (1999) have supported interpreting RMSEA values below 0.06 as acceptable. For CFI and TLI, values approximating .95 (or higher) were interpreted as indicative of good fit, and values between .90 and .95 were considered acceptable. In both EFA and CFA, we interpreted model comparisons by using the Satorra-Bentler chi-square difference test (SBΔx^2^) to examine differences in model fit and confirm which model had the better structure to define the concept under study (Satorra & Bentler, 2001).

#### Construct Validity

We tested the scale’s construct validity by connecting it with one component of physical self-concept that might be related to the perceived hockey competence construct through a more general construct of perceived sport competence (PSC). We added it as a dependent factor predicted by ice hockey perceived competence to the best model previously obtained in the last step of the validation process. According to Boateng et al. (2018), a regression measure is preferable to a simple correlation because it quantifies the association in meaningful units and facilitates the validity judgment. PSC was measured with three 5-point Likert-type items, extracted from the *Inventaire du Soi Physique*, the French version of Corbin’s Physical Self-Perception Profile (Ninot et al., 2000). Items were worded as follows: (a) *I find that all sports are easy for me, (b) I find that I’m good in all sports, and (c) I do well in sports*. Internal reliability of this subscale was measured using McDonald Omega coefficient (ω). Model fit indices from the first models were then compared to verify that perceived competence in hockey was associated with the global measure of perceived sport competence**.**

## Results

### Item Development

#### Domain Identification, Item Generation and Content Validity

Since no official definition of hockey competence exists, our proposed definition was: *All characteristics and aspects that directly or indirectly allow a player to perform well in ice hockey and have the potential to contribute to winning games*. We recruited 25 ice hockey experts (*M* years of experience = 14.50, *SD* = 1.40) at different competition levels. Once their responses were recorded, we compiled 251 statements. Once aggregated, these statements evolved into the initial 30 questions that were first reviewed by the experts. After three rounds of expert review, 3 of 4 or 75% of these experts reached a concensus that 29 items were satisfactory (see Appendix A). We kept the original 29 items for the next phase.

### Scale Development

#### Scale Pretesting and Test-Retest Reliability

As described above, we recruited a pilot sample composed of players from three U15 ice hockey teams (n = 42, 100% male; aged 14–16 years old). All players completed two waves of assessment seven-days apart. Correlations between Time 1 and Time 2 revealed that all items had a strong intraclass correlation coefficient (all > 0.87). Subsequently, there were no reported concerns about the clarity of items; and the time required to complete the questionnaire were reported by each participant.

#### Scale Validation

From an initial respondent sample of 1,111 players out of the 1188 initially contacted (93%), we deleted 91 duplicates and removed 250 goaltenders, whose competency skills were out of the scope of this study. The final EFA sample consisted of 770 players (98% male; *M* age = 14.78; *SD* = 1.60 years). This sample was considered sufficient for statistical power for a EFA (Soper, 2022). CFA, our participant sample was recruited six months following the initial phase. This second sample consisted of 204 competitive players out of a total of 240 initially recruited (85% response rate); these participants took part in a selection camp for the U16 male and U18 female national team and athletes were in preparation for a national competition (56% female, *M* age = 15.50, *SD* = 0.82 years).

### Item Reduction and Factor Extraction

As shown in Table 1, EFA results suggested that a 6-factor structure provided best fit indices. After analyzing for item factor loadings within the 6-factor model, we removed seven items that loaded on multiple dimensions and/or showed a factor loading < 0.4. Having reduced the initial 29 items to 22 items, we interpreted each factor according to item loadings as follows: Factor 1 - “Offensive Abilities” (4 items); Factor 2 - “Strength and Power” (4 items); Factor 3 - “Skating” (4 items); Factor 4 - “Tactics” (4 items); Factor 5 - “Coachability’’ (3 items); and Factor 6 -“Resilience” (3 items). McDonald’s omegas for each sub-scale were interpreted as satisfactory (ω_F1_ = 0.82; ω_F2_ = 0.83; ω_F3_ = 0.82; ω_F4_ = 0.75, ω_F5_ = 0.74, ω_F6_ = 0.74).

**Table 1.** Results from Exploratory Factor Analysis (n = 770): From a 1 to a 6-Factor Model.

Model	No. of Parameters	Chi-square	Degrees of Freedom	Model Comparison	SBΔx^2^ (df)
1-Factor	87	2 219.84	377	n/a	n/a
2-Factor	115	1 671. 63	349	M2 vs M1	548.21 (28)*
3-Factor	142	1 192.22	322	M3 vs M2	479.41 (27)*
4-Factor	168	768.92	296	M4 vs M3	423.30 (26)*
5-Factor	193	614.74	271	M5 vs M4	154.18 (25)*
6-Factor	217	466.30	247	M6 vs M5	148.44 (24)*
7-Factor	n/a	(Model 7 did not converge)			

* *p* < .001.

### Confirmatory Analyses: Tests for Dimensionality

As specified earlier, we conducted a CFA by considering the 6-factor model as the baseline for model estimation and comparing three CFA models. Model 1 was the CFA baseline model, in which we constrained each item on their corresponding dimensions (based on results from EFA). Model 2 was the extension of Model 1, in which we added associations between residual covariances. These inter-item covariances were added by interpreting the results from the LaGrange multiplier test. Moreover, we verified that these associations were theoretically plausible and would not bias the estimates of the model. Finally, Model 3 tested the hypothesis that perceived competence in ice hockey might be a second order construct. As shown in Table 2, (Model 1), the initial model suggested poor fit indices. This justified considering results from the Lagrange multiplier test, which suggested the addition of seven residual covariances. Model 2 displayed a significant improvement of model fit (SBΔx^2^ = 264.5 (7) *p* < .0001), and fit indices of Model 2 were still superior to Model 3, meaning that perceived competence in ice hockey is better conceptualized as a first order construct.

**Table 2.** Results from Confirmatory Factor Analysis (n = 204).

Model	Chi-square/df	CFI	TLI	RMSEA	SRMR
Model 1	372.45/215	0.867	0.794	0.065	0.071
Model 2	262.09/185	0.925	0.907	0.040	0.061
Model 3	275.50/194	0.921	0.906	0.044	0.050

Table 3 shows all item factor loadings on their corresponding dimensions and residual covariances. Among the seven residual covariances, five unique covariances were correlated. Two residual covariances came from items on the “Strength and Power” dimension: i1↔ i10, i1↔ i2. Two covariance between “Skating” indicators was added: i6↔ i7 and i9↔ i28, while another covariance was added among two “Resilience” indicators: i15↔ i29. Two (out of the nine) residual covariances involved items associated with different dimensions: i29↔ i11 referred to items that represented work ethic and relationships with coaches and i23↔ i28 referred to defensive actions, such as playing well defensively and being good at backchecking. All factors were significantly correlated (coefficients between 0.436 and .684, all at *p* < .01).

**Table 3.** CFA Results: Factor Loadings and Inter Factor Correlations of the Self-Perceived Ice Hockey Competence Scale (Standardized Coefficients; 22-Item Version (n = 204)).

Item: *As a player, I…*	F1	F2	F3	F4	F5	F6	Covariances
Factor 1: Offensive abilities	—	.576	.656	.629	.575	.436	
2. *have an accurate shot*	0.590						
3. *am good in stickhandling*	0.663						
20. *have good offensive creativity*	0.713						
24. *am efficient in offensive zone*	0.755						
Factor 2: Strength and power	—	—	.578	.692	.580	.483	
1. *have a powerful shot*		0.609					10, 2*
8. *am involved physically*		0.683					
10. *am physically strong*		0.621					
16. *win my “one-on-one” battles*		0.834					
Factor 3: Skating	—	—	—	.690	.506	.572	
*6. am a fast skater*			0.672				7
7. *have good skating agility*			0.675				
9*. have good endurance-stamina*			0.661				
28. *am good at back checking*			0.735				9
Factor 4: Tactics	—	—	—	—	.832	.660	
4. *make accurate passes to my teammates*				0.640			
19*. take good decision without the puck*				0.720			
21. *apply play systems efficiently*				0.717			
23. *am efficient in defensive zone*				0.666			28*,24
Factor 5: Coachability	—	—	—	—	—	.684	
11. *have a good work ethic*					0.702		29*
12. *see coach’s comments as opportunity to improve*					0.652		
13. *am a leader to my teammates*					0.637		
Factor 6: Resilience	—	—	—	—	—	—	
15. *accept my role within the team*						0.745	29, 11*
25. *stay confident even if playing time is diminished*						0.591	
29. *am not easily discouraged, even in difficult situation*						0.790	

*Note:* All coefficients significant at *p* < 0.01; *covariance that involves items from different dimensions.

### Construct Validity

**Figure 2.**
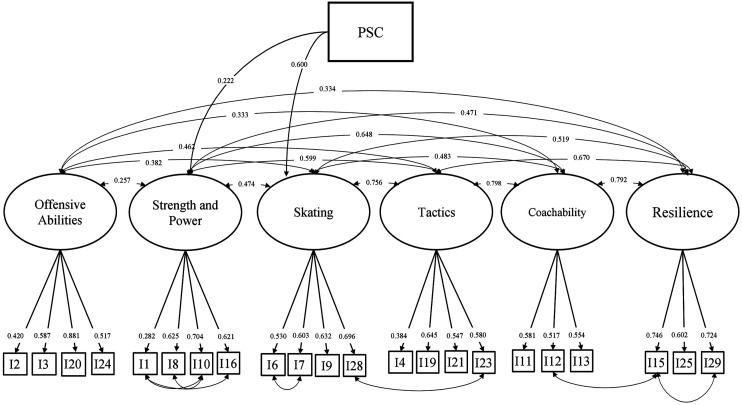
Structural Equation Model of Perceived Competence in Ice Hockey.

The subscale used to assess perceived sport competence (PSC; Ninot et al., 2000) showed very good psychometric properties (ω = .94). The final step was to assess construct validity by introducing a global measure of PSC as a dependent variable. We modeled this association by predicting PSC from Model 2 to see which of the six dimensions of perceived hockey competence would be related to global PSC. Results displayed acceptable fit indices: x^2^_(245)_ = 334.17, *p* < .001, RMSEA = 0.040, CFI = 0.931, TLI = 0.917; SRMR = 0.060. Two of the six perceived ice hockey competence constructs displayed significant regression paths towards PSC: β_skating_ = .600, *p* < .001; β_Strength and Power_ = .222, *p* = 0.01. The associations between the global measure of PSC factor and the “Offensive Abilities” (β = 0.061), “Power and Strength” (β = 0.092) and “Resilience” (β = −0.001) were not significant (*p* > .25). Total explained variance for global PSC was: R^2^ = 0.72. The final model with the introduction of the PSC variable is shown in Table 2. (Figure 2)

## Discussion

In this three-phase research project, we created a new instrument for assessing young ice hockey players’ self-perceived sport competence in ice hockey. Considering the multifaceted aspects of ice hockey, several items were related to physical involvement, skating, and tactics, which are all important to ice hockey performance. In addition, other items addressed psychological competence (e.g., resilience, leadership, and interpersonal relationships) was deemed relevant (Montgomery et al., 2004). We expected that competence in ice hockey would be multidimensional as was suggested in past research (Hristov, 2017), and the final set of items in this survey overview those player competencies that are required to excel in ice hockey and ensure that the survey is reliable and simple to complete.

The first phase of this research involved item creation with help from ice hockey experts. Next, we validated this process by assessing the questionnaire’s factorial validity, confirming that it is an effective tool for assessing self-perceived competence of adolescent ice hockey players. Consistent with our initial hypothesis, perceived competence was found to be multidimensional. Although its six dimensions are a higher number than found in a survey developed for soccer (Forsman et al., 2016), we expected that ice hockey complexity might have multifaceted demands (Fait et al., 2011). We considered our 10 hockey experts’ suggestions to consider psychological aspects of ice hockey competence. Our first factor, Offensive Abilities, seems purely related to technical skills contributing to offensive production. Strength and Power, a key aspect of ice hockey performance, has been historically important, with physical implications that are important in the minds of ice hockey players and other stakeholders (Guenter et al., 2019). Our third factor, Skating ability, was defined as a key dimension for hockey competencies a crucial element that distinguishes ice hockey from virtually every other sport and has become even more important in fast-paced modern team play (Bracko, 2001). Tactics, requires “*read-and-react*” skills related to reading systems and playing with and without the puck. Coachability is related to Bucci’s et al. (2012) description of player leadership skills and the ability to foster positive social interactions that are increasingly more important in team sports as the season advances (Duguay et al., 2020). Finally, our sixth factor, Resilience, is the ability to face adversity and challenging situations, it is critical to a player’s well-being, as it helps to limit anxiety present in major games and improves self-esteem (Kaplánová, 2019; Stanbulova et al., 2017). For athletes to develop strong perceptions of their psychological assets is crucial to ice hockey success and might be transferable to other aspects of life on a longer-term basis as suggested within the *Personal Assets Framework* (Côté et al., 2016).

### Limitations and Directions for Further Research

A limitation of this study is that we conducted it among players who evolved in ice hockey organizations under similar developmental conditions and who may have a shared understanding of the items on the questionnaire. Future researchers will need to determine whether other variant sub-populations (e.g, female ice hockey players and those from other provinces) have sufficiently similar hockey backgrounds for this instrument to be applicable to them. Our almost exclusive reliance on male participants was a particular limitation. Future researchers should consider girls’ opinions about their perceived competence in ice hockey and conduct a separate analysis of whether this survey is gender invariant. Our participants were exclusively French-speaking, and it will be important to adapt this survey to other languages and cultures. Future studies should also ensure the validity and structural invariance of the questionnaire among English-speaking players (e.g., other Canadian provinces) and even among players from other cultures in which ice hockey is a well-established sport (e.g., Finland, Sweden, Swiss, Germany, Czech Republic, Russia, and the United States) (Hambelton et al., 2004). While our measurement of test-retest reliability was done before we established scale validation, we changed no questions after this point, and we do not believe that this decision adversely impacted scale quality. Nevertheless, future investigators should cross-validate the test-retest reliability of the questionnaire to ensure its reliability. In addition, since the validation process was done on a single sample due to the sample size required to complete the analyses, it would be beneficial to distribute the questionnaire to another group of players to fully ensure its validity. In terms of future development, criterion validation of the questionnaire dimensions (e.g., skating abilities, game performance, coach evaluation) might provide additional knowledge for this instrument’s development.

## Conclusion

The measurement of sport-specific self-perceived player competence has been raised by others as a weakness in the existing literature for adolescent hockey players, and we attempted to address that gap in the literature by developing a new valid and reliable instrument would help players and coaches improve their understanding and enhance communications between the parent-athlete-coach triad (Preston et al., 2020; Smoll et al., 2011). We developed and reported here the Self-Perceived Ice Hockey Competence Scale to allow researchers and sport leaders to assess players’ perceived competence and use that information to create a healthy environment and motivational climate for fostering players’ developmental improvements.

This instrument can now be used for players’ self-evaluation at specific stages of their development to inform, for example, whether (a) elite players have significantly more positive self-perceptions than those playing on an outdoor rink, or, similarly, (b) the perceptions of varsity players differ from those of major junior or professional players of the same age. Additonally, this instrument now enables the development of cross-sectional and prospective research that compares the outcomes of sport development models.

Our finding of a relationship between some dimensions of perceived competence in ice hockey and a global measure of Perceived Sport Competence (PSC;Estevan & Barnett, 2018) is reassuring regarding the validity of this new measure. The regression paths we identified between perceived competence in ice hockey and, particularly, the Skating and Strength and Power factors relate to the physical condition of the player and move the scale beyond a narrow focus on technical aspects of skating (despite their separate importance) to a broad sport-specific tool.

## Appendix

**Table:** **Appendix A.** Intraclass Correlation Coefficients (ICC) (with Pilot Sample: n=42).

*Item*	*M ± SD T1*	*M ± SD T2*	*ICC*
*1. Je lance avec puissance.*	3.75 ± 1.01	3.86 ± 0.89	0.945 **
*2. Mes tirs au but sont précis.*	3.75 ± 0.70	3.79 ± 0.74	0.910**
*3. J’excelle dans le maniement de la rondelle.*	3.6 ± 0.69	3.64 ± 0.68	0.878**
*4. J’effectue des passes précises à mes coéquipiers.*	4.25 ± 0.52	4.21 ± 0.63	0.981**
*5. J’ai de la facilité à recevoir les passes de mes coéquipiers.*	4.11 ± 0.63	4.14 ± 0.65	0.987**
*6. Je patine rapidement.*	4.00 ± 0.72	4.07 ± 0.72	0.995**
*7. Je me déplace avec agilité sur la patinoire.*	3.89 ± 0.83	3.82 ± 0.77	0.995**
*8. Je m’implique physiquement sur la glace.*	4.14 ± 0.89	4.11 ± 0.92	0.995**
*9. Je suis endurant au niveau cardiovasculaire.*	4.11 ± 0.69	4.25 ± 0.75	0.975**
*10. Je suis fort physiquement.*	4.00 ± 0.77	4.04 ± 0.84	0.995**
*11. J’ai des habitudes de travail qui m’aide à performer.*	4.43 ± 0.63	4.54 ± 0.69	0.989**
*12. Je vois les critiques de mon entraineur comme une occasion de m’améliorer.*	4.11 ± 0.74	4.04 ± 0.79	0.989**
*13. J’influence positivement mes coéquipiers.*	4.36 ± 0.56	4.25 ± 0.65	0.985**
*14. Je suis à mon meilleur dans les compétitions importantes.*	4.32 ± 0.67	4.29 ± 0.66	0.987**
*15. J’accepte le rôle que l’entraineur me donne au sein de l’équipe.*	4.25 ± 0.75	4.43 ± 0.74	0.985**
*16. Je gagne mes batailles à 1 contre 1.*	3.96 ± 0.69	3.93 ± 0.72	0.981**
*17. Je me sacrifie pour mon équipe.*	4.25 ± 0.80	4.11 ± 0.83	0.989**
*18. Je prends des décisions intelligentes avec la rondelle.*	4.25 ± 0.70	4.29 ± 0.71	0.990**
*19. Je prends des décisions intelligentes sans la rondelle.*	4.07 ± 0.60	4.04 ± 0.64	0.970**
*20. Je suis créatif sur la glace.*	3.82 ± 0.72	4.11 ± 0.83	0.985**
*21. Je peux appliquer les systèmes de jeux sans problème.*	4.43 ± 0.63	4.25 ± 0.65	0.989**
*22. Je me positionne dans les espaces libres pour recevoir des passes.*	4.00 ± 0.77	4.03 ± 0.74	0.987**
*23. Je suis efficace en zone défensive.*	4.11 ± 0.63	3.89 ± 0.79	0.987**
*24. Je suis efficace en zone offensive.*	4.07 ± 0.60	4.03 ± 0.64	0.993**
*25. Je reste confiant même si mon temps de jeu diminue.*	3.64 ± 0.91	3.82 ± 0.98	0.991**
*26. J’anticipe le jeu de mes adversaires.*	4.00 ± 0.67	3.96 ± 0.58	0.993**
*27. Je protège bien la rondelle, les adversaires ne m’enlèvent pas facilement la rondelle.*	4.07 ± 0.72	4.04 ± 0.74	0.974**
*28. Je suis bon dans les replis défensifs.*	4.21 ± 0.63	4.18 ± 0.70	0.993**
*29. Quand j’ai des difficultés je ne me décourage pas ou pas longtemps.*	4.11 ± 0.79	4.11 ± 0.74	0.988**

M: Mean; SD: standard deviation ***p* < 0.001.
